# Relationship of moderate and low isometric lumbar extension through architectural and muscular activity variables: a cross sectional study

**DOI:** 10.1186/1471-2342-13-38

**Published:** 2013-11-19

**Authors:** Antonio I Cuesta-Vargas, Manuel Gonzalez-Sanchez

**Affiliations:** 1 School of Clinical Sciences, Faculty of Health, Queensland University of Technology (QUT), Victoria Park Road, Kelvin Grove QLD 4059, Australia; 2 Department of Physiotherapy, Faculty of Health Sciences, University of Malaga, 29071, Málaga, Spain

**Keywords:** Ultrasonography, Surface electromyography, Thickness, Pennation angle, Erector spinae

## Abstract

**Background:**

No study relating the changes obtained in the architecture of erector spinae (ES) muscle were registered with ultrasound and different intensities of muscle contraction recorded by surface EMG (electromyography) on the ES muscle was found. The aim of this study was analyse the relationship in the response of the ES muscle during isometric moderate and light lumbar isometric extension considering architecture and functional muscle variables.

**Methods:**

Cross-sectional study. 46 subjects (52% men) with a group mean age of 30.4 (±7.78). The participants developed isometric lumbar extension while performing moderate and low isometric trunk and hip extension in a sitting position with hips flexed 90 degrees and the lumbar spine in neutral position. During these measurements, electromyography recordings and ultrasound images were taken bilaterally. Bilaterally pennation angle, muscle thickness, torque and muscle activation were measured. This study was developed at the human movement analysis laboratory of the Health Science Faculty of the University of Malaga (Spain).

**Results:**

Strong and moderate correlations were found at moderate and low intensities contraction between the variable of the same intensity, with correlation values ranging from 0.726 (Torque Moderate – EMG Left Moderate) to 0.923 (Angle Left Light – Angle Right Light) (p < 0.001). This correlation is observed between the variables that describe the same intensity of contraction, showing a poor correlation between variables of different intensities.

**Conclusion:**

There is a strong relationship between architecture and function variables of ES muscle when describe an isometric lumbar extension at light or moderate intensity.

## Background

An in-depth study of the behaviour of musculoskeletal structures allows access to a wealth of information that could be very useful for understanding the response of these structures to different situations or stimuli [1,2]. To that end, many studies have resorted to investigating architectural and functional variables in isolation [3-7] and integrating both types of variable [8-10]. Muscle fibre pennation angle (defined as the angle that creates muscle fibers with respect to the muscular aponeurosis [11]) and muscle thickness (defined as the shortest distance between surface and deep aponeurosis of muscle [11]) are two variables of muscle architecture, which have been utilised in various studies as indicators of the force generated by the muscle during contraction [10,12]. Many studies have been published in which at least one of these two variables was considered to describe muscle behaviour after a given stimulus [8-10,12-14], but the relationship between ultrasonographic and electromyographic variables is somewhat controversial. Some studies have shown a strong relationship between them, while others have found a low ratio [8-10,12].

In the field of biomechanics, US (ultrasound) has become a widely used tool to describe changes in muscular architecture produced during muscle contraction [4,14-16]. In recent years, several studies have been published using ultrasound to examine the changes in the muscle ES (erector spinae) in different situations [7,8].

In addition, surface electromyography has been used in different studies related to both muscle strength and muscle activation levels [14,17-19]. Some studies have made use of surface electromyography to analyse the level of activation of the paraspinal musculature with different types of contraction [17,18]. Its ease of use has allowed it to be a widely used tool in research and with great potential in clinical practice.

No study relating the changes obtained in the architecture of ES muscle were registered with ultrasound and different intensities of muscle contraction recorded by surface EMG (electromyography) on the ES muscle was found. It is important to study the activation together with the contraction (architectural variables) of the ES muscle for several reasons: the anatomical characteristics of the ES are clinically important in patients with LBP (low back pain), since it is actuated and inserted into the lumbar spine [20-22]. In addition, ES muscle is a tonic muscle [20,21]. The average intensity of contraction is usually moderate or light, necessary to maintain an upright posture and to assist the movement of lateral flexion, rotation and lumbar extension [23]. This study chased two objectives. To describe the response of the ES muscle during maximal, moderate and light isometric contraction, analysing architecture and function variables, obtained by ultrasound and surface electromyography. The second is to analyse analyse the relationship between architecture and functional variables during moderate and light contractions of the ES muscle using normalized values from MVC.

Our hypothesis was that there is a significant relationship in the response generated by the ES muscle during moderate and light isometric trunk and hip extension in a sitting position with hips flexed 90 degrees and the lumbar spine in neutral position between the seven variables (muscle activation (EMG), muscle thickness and pennation angle (right and left side) and torque) studied in the three contraction intensities.

## Methods

### Design

Cross-sectional study. The independent variable was percentage of maximal extensor force and dependent variables were level of muscle activation, ES thickness and pennation angle.

### Participants

**Figure 1:**
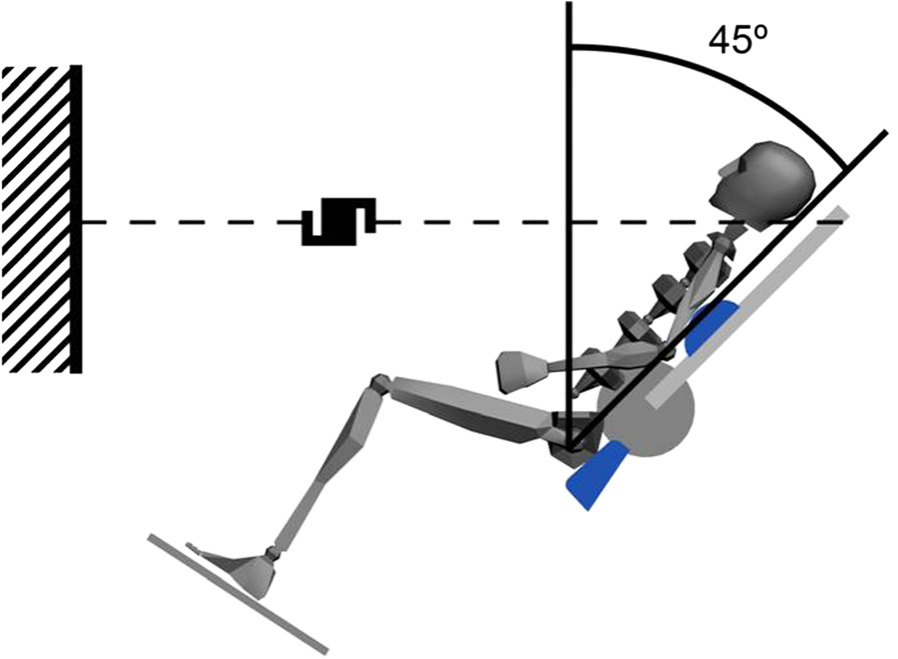
Scheme of the participants position and ejecution of the gesture analized.

46 subjects (52% men) with a group mean age of 30.4 (±7.78) participated in this study. In this trial, muscle activation (measured with surface electromyography) and architecture variables of the ES muscle (obtained with ultrasound) were measured during isometric lumbar extension while performing moderate and light isometric trunk and hip extension in a sitting position with hips flexed 90 degrees and the lumbar spine in neutral position (Figure 1). This study was developed at the human movement analysis laboratory of the Health Science Faculty of the University of Malaga (Spain). Ethics approval was attained from the ethics committee of the University of Malaga and all subjects gave informed consent following the guidelines of the Helsinki Declaration of 1964 which sets out ethical principles for all human inquiry and has been upgraded in successive meetings of the World Medical Association [24].

### Procedure

During the test, all participants wore sneakers and the foot was supported on a platform. The dynamometer was adjusted so that the axis of rotation was at the height of L_5_ - S_1_. The hip and thigh were fixed to the chair using belts, so that movement of the thigh was minimised. The knee and the hip were 90° flexed to reduce the tension supported by the hamstring muscles (Figure 1).

### Maximal isometric force registration

Maximum isometric force was recorded by a load cell (RealPower, Globus, Italy), which was located between two chains. One chain was fixated to the wall and the other to the measurement apparatus (Figure 1). The subject developed an isometric contraction at 45 grades from the vertical (Figure 1).

### Electromyography

Two bipolar surface electromyography electrodes (Datalog Biometrics, England; amplifier bandwidth: 20–450 Hz, common mode rejection ratio: 60 Hz (dB) > 96, typically 110 dB; input impedance 1015 Ohms; sampling frequency: 1000 Hz) with an inter-electrode distance of one centimetre were place on the skin surface 3 cm lateral from the spinous process of L_3_-L_4_. Electromyographic value was taken from the difference between the maximum and minimum registration. Prior to this calculation, all recorded signals were passed 20 HZ low pass Butterworth filter to remove high-frequency noise from the sample. Datalink software 3.0 managed the process of acquisition of values and treatment records.

### Ultrasound registration

**Figure 2:**
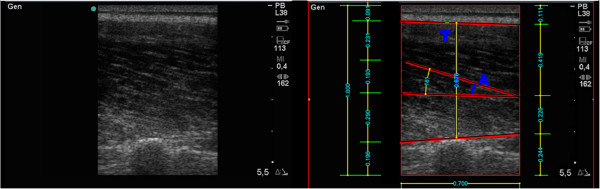
**Example architecture variables measure from ultrasound image. A:** Pennation Angle. **T:** Muscular Thickness.

The SonoSite Mod Titan ultrasound system was used in this study. It has a head 5 cm in width, and was placed immediately below the EMG sensors, placed parallel to the longitudinal axis of the muscle. From this perspective, and at a depth of 5.5 cm, it was possible to obtain images to calculate both the pennation angle and the thickness of the ES. Figure 2 shows an example of a measure of this two muscle architectural variables (pennation angle and muscle thickness) from an ultrasound image. The ultrasonographer experience is higher than 5 years, ensuring proper collection of the ultrasound images.

For the analysis of all architectural variables, the clearest image was chosen to assure the right selection of the reference points of each measured parameter. Ultrasound is an instrument that has shown moderate to excellent reliability for the paraspinal musculature with intraclass correlation values ranging between 0.72 and 0.98 [25]. This tool has the disadvantage of relying on the skill of the operator when making a measurement. However, for the ES muscle, one study has shown that interobserver reliability ranged between 0.900 and 0.948, while intraobserver values varied from 0.938 and 0.962 [7].

### Experimental protocol

Before initiating the protocol for this study, participants filled out four questionnaires regarding their general health status (SF-12 (Short Form 12) [26,27], quality of life (EQ 5D (EuroQoL 5D) [28,29], level of disability (RMQ (Roland Morris Questionnaire) [30,31] and Orebro Musculoskeletal Pain Questionnaire (OMPQ) [32,33].

Figure 1 shows the schematic position of participants into pre-calibrated machine. Each subject made several attempts without charge to find the most comfortable position to developed the movement. Then the load cell mentioned previously record the torque of the isometric lumbar extension at 45 degrees from the vertical (90 degrees between hips and trunk flexion with the lumbar spine in neutral position). Two straps placed one on another in the hip and thigh, to ensure maintenance of the neutral position of the lumbar spine during movement execution. Before beginning the protocol, the correct subject position was confirmed using an electronic goniometer.

Each subject performed three thrusts during five seconds with a break of 90 seconds between trials. The peak force recorded was considered the maximum force that the participant could exert for that movement.

From this measurement as a reference, each subject then performed three isometric lumbar extensions for 5 seconds for each intensity: light (33% MVC), moderate (66% MVC) and maximal (100% MVC) (9 repetitions in total). The rest between each repetition was 90 seconds.

During these measurements, electromyography recordings and ultrasound images bilaterally were and torque obtained following the methodology explained above. From the ultrasound images, the architectural variables (pennation angle and muscle thickness) were obtained while the EMG recording allowed for calculating the value of muscle activity during isometric contraction at 33% (light), 66% (moderate) and 100% MVC (maximal voluntary contraction). The values of the 100% MVC were used to normalized the moderate and light isometric contractions.

Thus, the variables considered for analysis in this study were: Moderate contraction: pennation angle right side (AR Mod), pennation angle left side (AL Mod), muscle thickness right side (TR Mod), muscle thickness left side (TL Mod), muscle activation right side (EMG R Mod), muscle activation left side (EMG L Mod) and torque (Torque Mod). Light contraction: pennation angle right side (AR Light), pennation angle left side (AL Light), muscle thickness right side (TR Light), muscle thickness left side (TL Light), muscle activation right side (EMG R Light), muscle activation left side (EMG L Light) and torque (Torque Light).

### Data analysis

The average value of muscle activation and torque during relative isometric contractions (66% and 33%) were considered as the measure of these variables during each contraction. Muscle thickness and pennation angle were measured following an adapted procedure described by Hodges et al. [34]. Thickness was measured as the distance between the superficial and deep aponeuroses. Pennation angle was measured between a parallel aponeurosis line and the line of the clearest fascicle as the positive angle.

Data analysis was conducted in two parts. We performed a descriptive analysis of the results. The pool of data of EMG and US signals were normalized using package rank of free software R. Spearman bivariate correlation between architectural and functional variables of the ES muscle. These analyses were calculated using normalized data when performing moderate and light isometric lumbar extension. The interpretation of correlation coefficients used was: r < 0.49, poor correlation, 0.50 > r < 0.74, moderate correlation, r > 0.75, strong correlation [35].

The Statistical Package for the Social Sciences (SPSS) (version 17.0 for Windows, Illinois, and USA) was used to perform data analysis.

## Results

Table 1 shows the descriptive data of the sample obtained from subjective questionnaires. The results show the minimum, maximum, average and standard deviation of the characteristic of sample. Table 2 shows the values that describe the sample when they performed the tests at maximal, moderate and light intensities. It shown absolute values for all intensities and normalized for moderate and light intensities, with the standard deviation and the 25 and 75 percentile. Considering that we investigated a group of young adult subjects whose anthropometric indices were normal for their perception of quality of life (EQ 5D and EQ VAS), general health (SF-12 physical component state and SF-12 mental component state), and level of musculoskeletal disabilities, both in general (OMPQ) and in the back region (RMQ).

**Table 1:** Descriptive statistics data obtained from subjective questionnaires

	**Minimum**	**Maximum**	**Mean**	**SD.**
**Age (years)**	22	50	30.39	7.79
**Weight (Kg)**	43	176	73.59	21.20
**Height (m)**	154	193	170.52	16.93
**BMI (Kg/m**^**2**^**)**	17.7	30.4	23.71	3.16
**EQ 5D**	0.61	1.00	0.92	0.10
**EQ VAS**	49	100	79.76	11.78
**SF-12 PCS**	22.81	65.96	51.77	8.93
**SF-12 MCS**	18.92	62.24	49.14	8.25
**OMPQ**	2	116	47.22	29.10
**RMQ**	0	9	1.43	2.33

EQ 5D: EuroQoL 5 dimensions.

EQ VAS: EuroQoL VAS.

SF-12 PCS: Physical Component State.

SF-12 MCS: Mental Component State.

ÖMPQ: Örebro Musculoskeletal Pain Questionnaire.

RMQ. Roland Morris Questionnaire.

**Table 2:** Descriptive statistics data obtained from functional tests

**Functional and US variables**	**25 percentile**	**75 percentile**	**Mean**	**SD**
Torque 100 (N · m)	46.41	76.67	**65.67**	20.39
Torque 66	32.75	55.33	**46.55**	14.70
Torque 33	19.43	31.59	**26.85**	8.31
AR Max (°)	12.00	14.00	**12.83**	1.24
AR Mod	8.52	9.79	**9.15**	0.93
AR Light	4.93	5.85	**5.39**	0.60
AL Max	12.00	14.00	**12.83**	1.24
AL Mod	8.31	9.61	**8.95**	0.89
AL Light	4.77	5.72	**6.31**	7.30
TR Max (mm)	36.28	42.43	**39.16**	5.59
TR Mod	26.74	31.18	**29.18**	4.18
TR Light	17.81	21.78	**19.91**	2.87
TL Max	35.63	45.38	**40.27**	6.73
TL Mod	25.06	32.25	**28.82**	5.28
TL Light	14.95	19.84	**17.08**	2.95
MVC R (mV)	353.50	748.50	**563.59**	227.24
Mod VC R	245.53	531.66	**402.31**	161.91
Mod Light R	149.10	324.26	**239.84**	95.82
MVC L	386.00	790.50	**590.63**	253.39
Mod VC L	276.00	534.25	**428.74**	195.72
Mod Light L	183.75	349.25	**284.17**	139.22
TR Mod Norm (%)	68.87	73.66	**71.53**	2.60
TL Mod Norm	69.11	74.12	**71.41**	3.10
AR Angle Mod Norm	69.07	73.86	**71.359**	2.65
AR Left Mod Norm	67.48	71.69	**69.79**	2.61
Mod VC R Norm	69.14	74.46	**71.46**	3.16
Mod VC L Norm	68.28	73.33	**71.18**	2.67
Torque Mod Norm	68.19	73.64	**70.85**	3.06
TR Light Norm	40.17	44.10	**42.08**	2.50
TL Light Norm	40.91	43.96	**42.38**	2.24
AR Angle Light Norm	40.12	43.64	**41.98**	2.19
AR Left Light Norm	39.70	42.26	**41.09**	1.92
Light VC R Norm	40.94	43.95	**42.65**	2.26
Light VC L Norm	41.81	44.35	**42.77**	1.95
Torque Light Norm	39.15	43.20	**41.15**	2.37

AR Max = Angle Right Maximum; AR Mod = Angle Right Moderate Intensity; AR low = Angle Right low Intensity; AL Max = Angle Left Maximum; AL Mod = Angle Left Moderate intensity; AL low = Angle Left low intensity; Thick R Max = Thickness Right Maximum; Thick R Mod = Thickness Right Moderate; Thick R low = Thickness Right low; Thick L Max = Thickness Left Maximum; Thick L Mod = Thickness Left Moderate; Thick L low = Thickness Left low; MVC R = Maximal; Voluntary Contraction Right; Mod VC R = Moderate Voluntary Contraction Right; Light VC R = low Voluntary Contraction Right; MVC L = Maximal Voluntary Contraction Left; Mod VC L = Moderate Voluntary Contraction Left; Light VC L = low Voluntary Contraction Left; Norm: Normalized values.

Table 3 shows the relationship between the architectural and function variables. A strong significant correlation between data recorded for each intensity when the subjects performed moderate and light contraction were observed. Specifically, the correlation values observed during a moderate intensity range between 0.922 (AL Mod - AR Mod) and 0.726 (Torque Mod - EMG L Mod). In turn, the correlations for a light intensity ranges from 0.923 (AL Light - AR Light) and 0.731 (EMG L Light - TR Light) (Table 3). However, the correlation between variables of different intensities shows a very poor value being the maximum value of -0.326 (AL Light - TL Mod).

**Table 3:** Correlation between architecture and functional variables

	**TR Mod**	**TL Mod**	**AR Mod**	**AL Mod**	**EMG R Mod**	**EMG L Mod**	**Torque Mod**	**TR Light**	**TL Light**	**AR Light**	**AL Light**	**EMG R Light**	**EMG L Light**	**Torque Light**
**TR Mod**	1													
**TL Mod**	**0.917†**	1												
**AR Mod**	**0.877†**	**0.890†**	1											
**AL Mod**	**0.831†**	**0.855†**	**0.922†**	1										
**EMG R Mod**	**0.897†**	**0.907†**	**0.876†**	**0.839†**	1									
**EMG L Mod**	**0.825†**	**0.900†**	**0.812†**	**0.776†**	**0.915†**	1								
**Torque Mod**	**0.884†**	**0.826†**	**0.903†**	**0.858†**	**0.816†**	**0.726**^**†**^	1							
**TR Light**	-0.264	-0.303*	-0.315*	-0.235	-0.272	-0.217	-0.237	1						
**TL Light**	-0.199	-0.234	-0.256	-0.144	-0.204	-0.121	-0.194	**0.913†**	1					
**AR Light**	-0.237	-0.333*	-0.280	-0.165	-0.323*	-0.246	-0.189	**0.779†**	**0.863†**	1				
**AL Light**	-0.251	-0.326*	-0.303*	-0.204	-0.309*	-0.213	-0.205	**0.776†**	**0.851†**	**0.923†**	1			
**EMG R Light**	-0.184	-0.214	-0.248	-0.143	-0.184	-0.109	-0.181	**0.861†**	**0.862†**	**0.904†**	**0.871†**	1		
**EMG L Light**	-0.219	-0.248	-0.314*	-0.220	-0.213	-0.208	-0.242	**0.731†**	**0.812†**	**0.746†**	**0.757†**	**0.901†**	1	
**Torque Light**	-0.130	-0.218	-0.214	-0.113	-0.223	-0.164	-0.146	**0.733†**	**0.852†**	**0.907†**	**0.864†**	**0.852†**	**0.774†**	1

T: thickness.

A: angle.

R: right.

L: left.

EMG: electromiography.

Mod: moderate.

Significance level:

* = 0.05.

† = 0.01.

The "bold details" attempts to facilitate the identification of significant correlations.

## Discussion

To our knowledge, this is the first study that has investigated the behaviour observed during isometric ES muscle contraction at different intensities followed by correlation analysis of the architectural and functional variables. Strong and moderate significant correlations were observed between the variables of the same intensities; however, the correlation between variables of different intensities was poor. While we have found strong correlations study on the architectural and functional variables of the ES during moderate and light lumbar isometric extension. Other trials have found a correlation in other trunk muscles [11,12,35,36].

The observed correlations between variables of the same intensity are strong, showing values ranging from 0.726 (Torque Mod - Mod EMG L) and 0.923 (AL Light - Light AR). Furthermore, when the same variable correlated to each side at the same intensity, in any case, the correlation value never falls below 0.9. These values demonstrate a clear relationship between architectural change measures and functional activation measures for the ES when develop a lumbar isometric contraction at moderate and light intensities. These results are consistent with other studies that have indeed found significant correlations between these variables in the abdominal muscles. John and Beith [36] published a good correlation value for the EO (external oblique), presenting a range of correlation between 0.63 and 0.94 during isometric trunk rotation. In addition, Hodges et al. [34] showed a correlation index for transverse and IO (internal oblique) ranging between 0.84 and 0.90, but found no obvious correlation for EO. Brown et al. [12] who investigated three muscles that compose the abdominal wall (internal oblique (IO) and external oblique (EO) and transverse), did not found a clear correlation between the muscle activation degree and changes in the muscles during isometric positions for shortening of these muscles.

A study presented by Dickx et al. [17] noted, a strong relationship between the architectural changes observed in the ES and multifidus muscles, as measured by magnetic resonance imaging recording the level of muscle activation by EMG. The correlation coefficients were 0.957 for the multifidus and 0.887 for the ES. It is very interesting to analyze how the higher correlation values were observed between the variables describing the isometric contraction at the same intensity. These results may be due to the fact that all participants changed the variables studied when performing moderate and light contraction. However, the level of variation within subjects at each contraction intensity analyzed was differently.

One study measured the thickness of the ES [6] and presented a mean value of 39.4 (± 4.2 mm) [6]. These values were quite different to those observed during light contraction (19.91 ± 2.87 and 17.08 ± 2.95, right and left, respectively). However, the values measured during moderate contraction (29.18 ± 4.18 and 28.82 ± 5.28, right and left, respectively) were much closer to the average found in this study. This difference in the measures would be due to the difference in the position of the subject at the time when measurements were taken. While our study investigated at 90 degrees of hips flexion with lumbar spine in neutral position, Masuda’s study [6] measured subjects at maximum extension. On the other hand, the muscle in our study was measured at maximal, moderate and light contraction, while in the study of Watanabe the muscle was measured in relaxation.

### Study limitation

The ultrasound measurements have proven to be very reliable when it comes to being registered, but there is a small margin of error that must be taken within the totality of the results presented. It would also be advisable to conduct the same study in other positions and intensities of contraction, so as to increase the knowledge on the behaviour of a muscle so important for the statics and dynamics of the lumbar spine.

## Conclusion

This study is the first to analysed the behaviour of the ES muscle from an architectural perspective while trying to find a functional relationship between the three variables considered (muscle thickness, pennation angle of the muscle fibres and muscle activation) during moderate and light isometric trunk and hip extension in a sitting position with hips flexed 90 degrees and the lumbar spine in neutral position.

It has been observed a strong correlation between the functional variables (EMG and torque) and architecture (pennation angle and muscle thickness) ES muscle when describing a moderate isometric lumbar extension and light. This strong correlation has been observed in the variables describing the gesture performed at the same intensity.

## Abbreviations

ES: Erector spinae; LBP: Lowback pain; EMG: Electromyography; SF-12: Short form 12; EQ 5D: EuroQoL 5D; RMQ: Roland Morris Questionnarire; OMPQ: Orebro musculoskeletal pain questionnaire; MVC: Maximal voluntary contraction; US: Ultrasound; SPSS: Statistical package for the social sciences; IO: Internal oblique; EO: External oblique.

## Competing interests

The authors state that no conflicts of interest have been reported by the authors or by any individual in control of the content of this article. This information has not been presented previously.

## Authors’ contributions

AI C-V participated in the conception and design of the study, in the data collection, analysis and interpretation of data and helped to draft the manuscript. M G-S participated in the data collection, analysis and interpretation of data and drafted the manuscript. All authors read and approved the final manuscript.

## Authors’ information

Antonio I. Cuesta-Vargas is Senior Lecturer in the University of Malaga, Manuel González-Sánchez is a PhD Candidate in the university of Malaga.

## Pre-publication history

The pre-publication history for this paper can be accessed here:

http://www.biomedcentral.com/1471-2342/13/38/prepub
